# Supplementary material for: An Endogenous Retrovirus Vaccine Encoding an Envelope with a Mutated Immunosuppressive Domain in Combination with Anti-PD1 Treatment Eradicates Established Tumours in Mice
Source: Viruses. 2023 Apr 6;15(4):926. doi: 10.3390/v15040926 (PMC10141008; doi:10.3390/v15040926)
Supplement: Supplementary file 1 [file viruses-15-00926-s001.zip › viruses-2254966-supplementary.pdf]

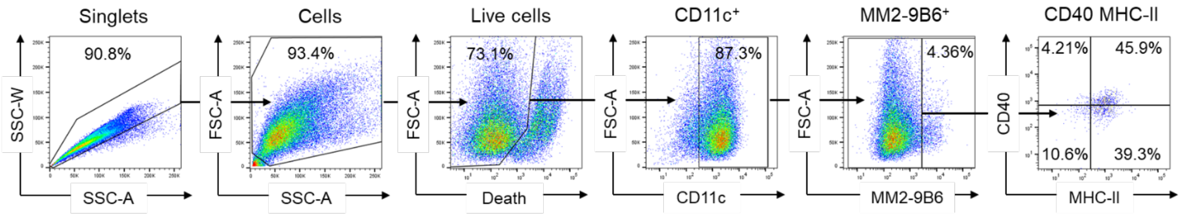

Figure S1. Gating strategy for the analysis of VLV transduced mouse BMDCs. Representation of the gating strategy to evaluate the expression of the MelARV Env target antigen (MM2-9B6^{+}) and DC maturation/activation markers (CD40^{+} and MHC-II^{+}) on the surface of mouse BMDCs (CD11c^{+}), 24 h after VLV transduction.

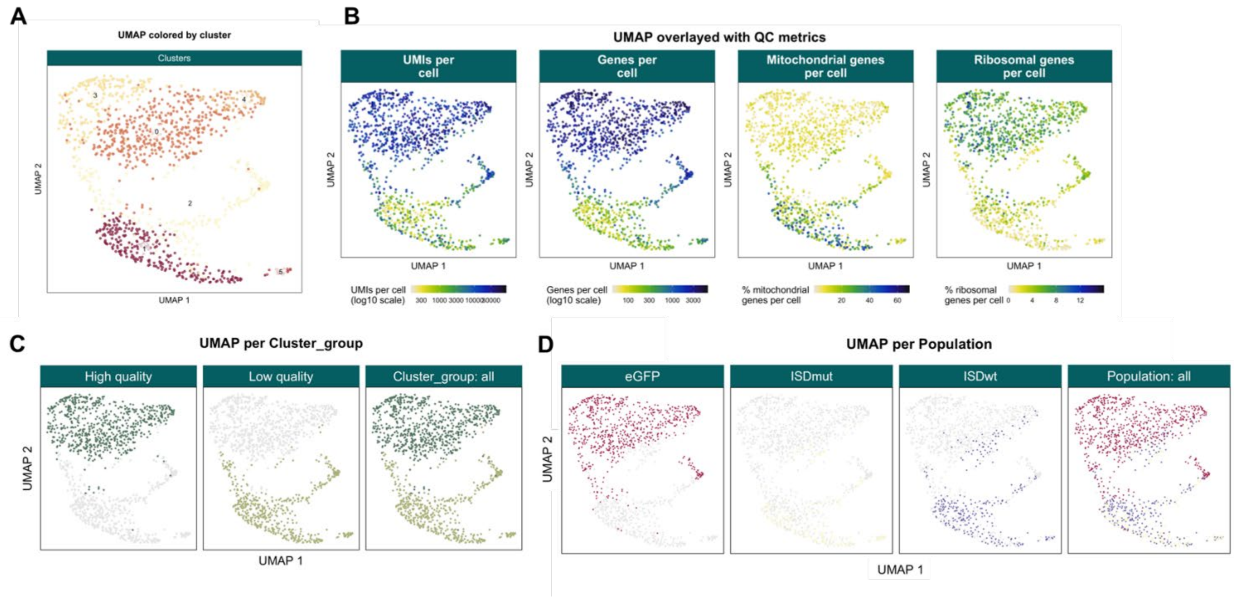

Figure S2. Single cell RNA sequencing analysis of VLV transduced mouse BMDCs. (Continued on the next page).

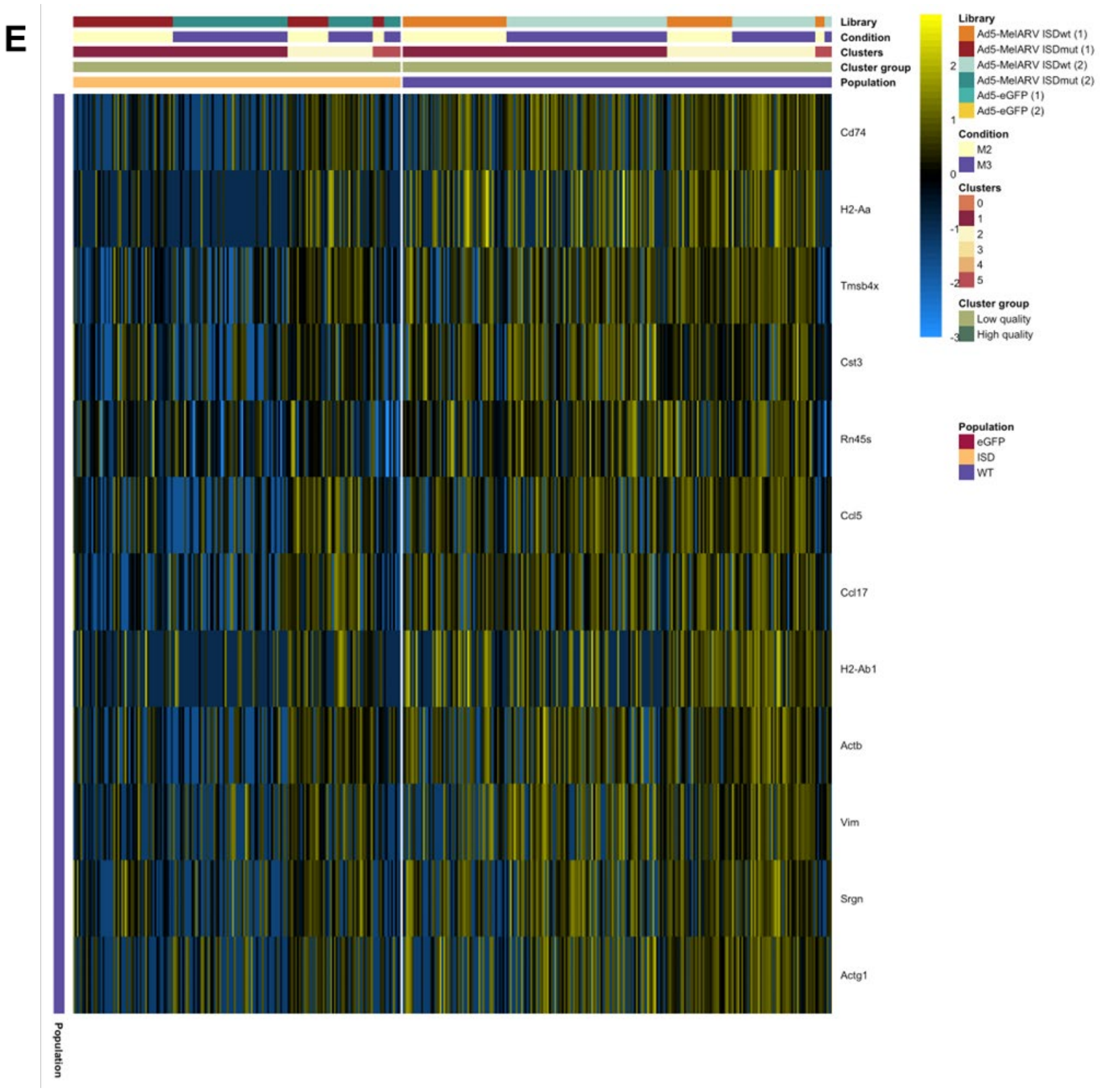

Figure S2. Single cell RNA sequencing analysis of VLV transduced mouse BMDCs. (**A**) UMAP showing 6 different clusters from single cell RNA sequencing analysis of mouse BMDCs transduced with either Ad5-eGFP, Ad5-MelARV ISDwt or Ad5-MelARV ISDmut. (**B**) Quality control of clustering. Observing separation by quality metrics, however regression of number of UMIs and mitochondrial content did not change the separation. (**C**) UMAP defining groups of high- and low-quality data. (**D**) UMAP showing clustering of different transduction groups. eGFP transduced BMDCs grouping differently from ISDmut and ISDwt. (**E**) Differential gene expression analysis between ISDwt and ISDmut populations. Only genes with an adjusted p value < 0.05 are shown.

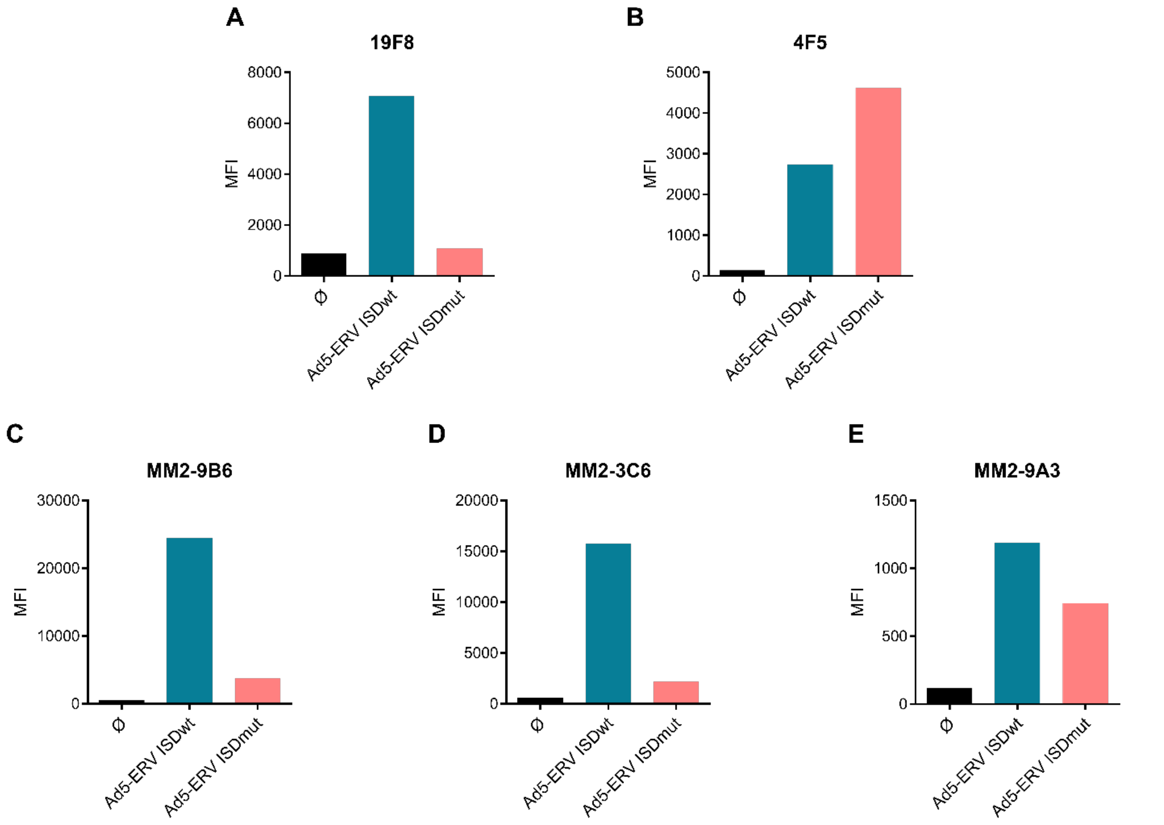

Figure S3. Detection of MelARV Env on the surface of transduced cells with Ad5-ERV ISDwt or ISDmut. Transduced Vero cells with Ad5 encoding MelARV Env wt or mut ISD sequence were surface stained with various anti-MelARV Env antibodies for the detection of MelARV Env: (**A**) 19F8 antibody targeting the ISD of p15E Env subunit, (**B**) 4F5 against p15E Env subunit, (**C**) MM2-9B6 antibody binding gp70 Env subunit, (**D**) MM2-3C6 antibody against gp70 Env subunit, **(E)** MM2-9A3 antibody targeting gp70 Env subunit. Env detection on the surface of transduced cells was analyzed by flow cytometry. Graph bars (N=1) show the mean fluorescence intensity (MFI) derived from the fluorescent-conjugated secondary antibodies.

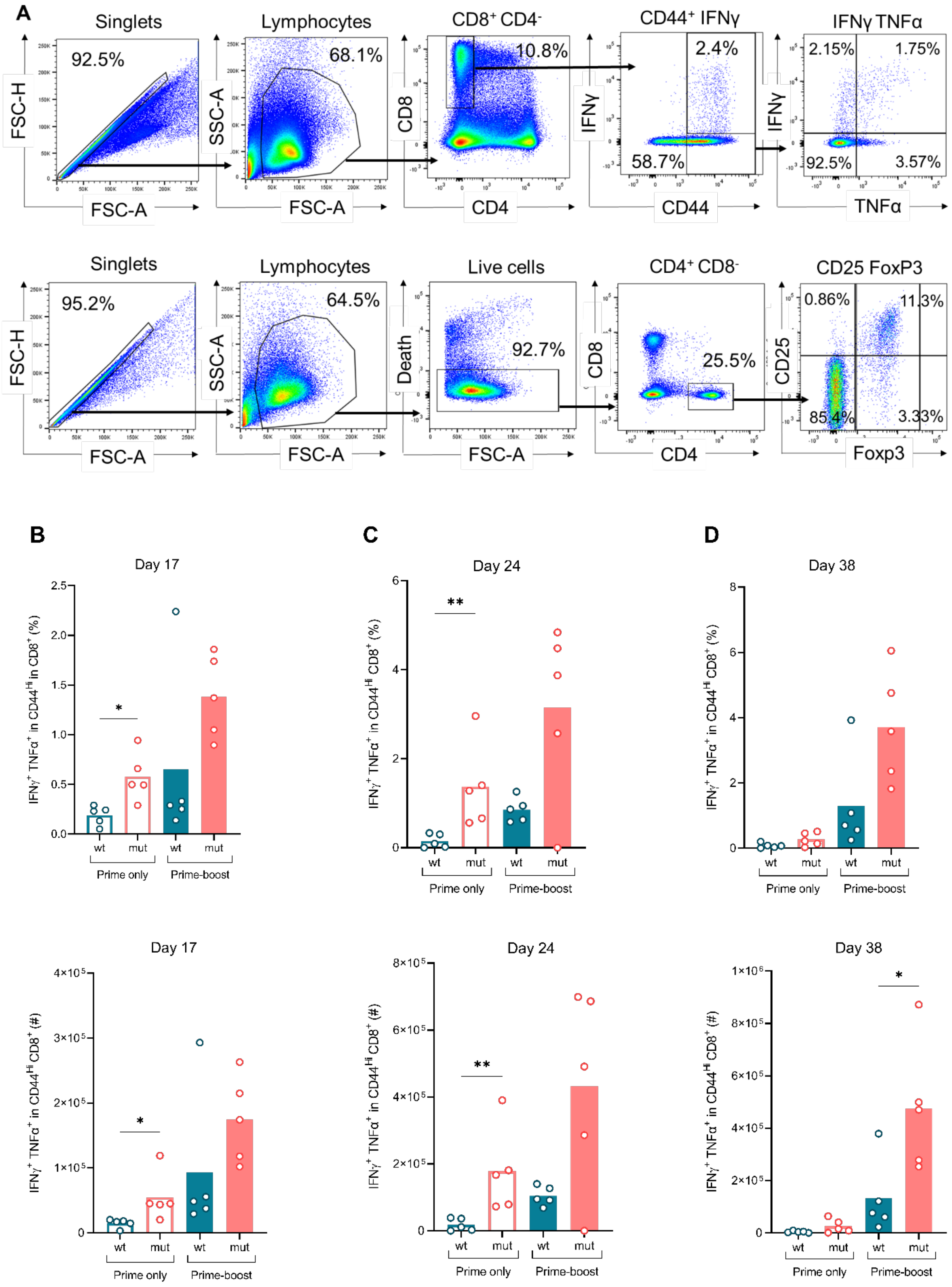
A
Singlets
92.5%
FSC-H
FSC-A
Lymphocytes
68.1%
SSC-A
FSC-A
CD8⁺ CD4⁻
10.8%
CD8
CD4
CD44⁺ IFNγ
2.4%
58.7%
IFNγ
CD44
IFNγ TNFα
2.15%
1.75%
92.5%
3.57%
IFNγ
TNFα
Singlets
95.2%
FSC-H
FSC-A
Lymphocytes
64.5%
SSC-A
FSC-A
Live cells
92.7%
Death
FSC-A
CD4⁺ CD8⁻
25.5%
CD8
CD4
CD25 FoxP3
0.86%
11.3%
85.4%
3.33%
CD25
Foxp3
B
Day 17
IFNγ⁺ TNFα⁺ in CD44Hi in CD8⁺ (%)
*
wt
mut
wt
mut
Prime only
Prime-boost
C
Day 24
IFNγ⁺ TNFα⁺ in CD44Hi CD8⁺ (%)
**
wt
mut
wt
mut
Prime only
Prime-boost
D
Day 38
IFNγ⁺ TNFα⁺ in CD44Hi CD8⁺ (%)
wt
mut
wt
mut
Prime only
Prime-boost
Day 17
IFNγ⁺ TNFα⁺ in CD44Hi CD8⁺ (#)
*
wt
mut
wt
mut
Prime only
Prime-boost
Day 24
IFNγ⁺ TNFα⁺ in CD44Hi CD8⁺ (#)
**
wt
mut
wt
mut
Prime only
Prime-boost
Day 38
IFNγ⁺ TNFα⁺ in CD44Hi CD8⁺ (#)
*
wt
mut
wt
mut
Prime only
Prime-boost

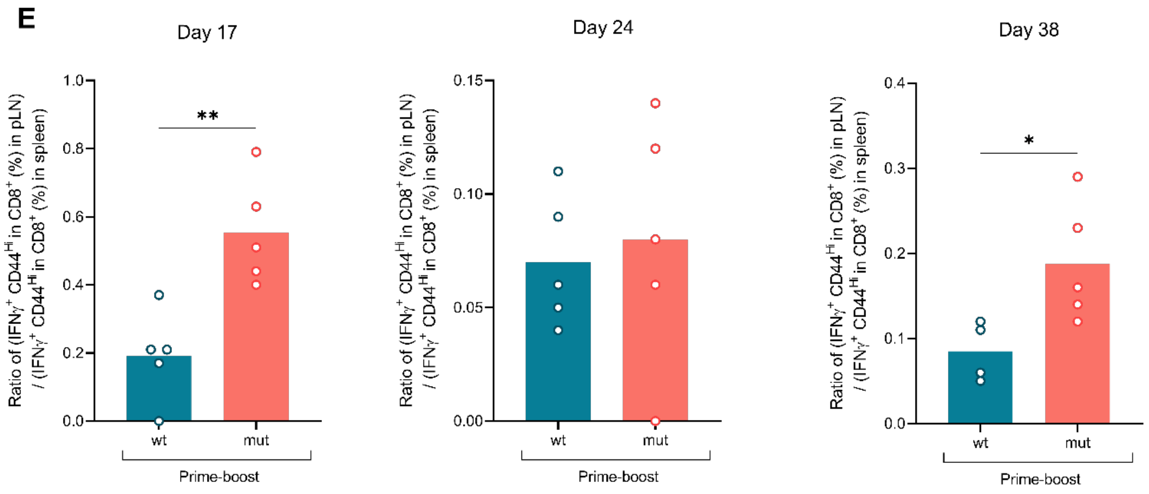

Figure S4. Immune profiling of spleen and pLN in vaccinated BALB/c mice. Mice were primed s.c. (right paw) on day 0 with either Ad19a/64-ERV ISDwt or Ad19a/64-ERV ISDmut. Half of the primed mice were boosted s.c. (left paw) on day 7, 14 or 28 with either the Ad5-ERV ISDwt or with the Ad5-ERV ISDmut vaccine. Ten days later, mouse spleens and left pLNs (vaccine draining LNs) were collected and restimulated with a H2-Ld-restricted MelARV Env T cell epitope (AH1 peptide). (**A**) Gating strategy of mouse splenocytes (also applicable to pLN) for the detection of IFNγ$^+$ TNFα$^+$ CD8$^+$ T cells (upper panel) and CD4$^+$ CD25$^+$ FoxP3$^+$ T_{regs} (lower panel) by flow cytometry. (**B-D**) Frequency (upper graphs) and total number (bottom graphs) of IFNγ$^+$ TNFα$^+$ in CD44^{Hi} CD8$^+$ T cells responding to MelARV Env AH1 peptide in the spleen of each mouse on day 17, 24 and 38 after prime. Day 17 was repeated in two independent experiments. (**E**) Ratio of the percentage of IFNγ$^+$ CD44^{Hi} in CD8$^+$ T cells in the left pLN (local immunity) and the spleen (systemic immunity). In this figure, one pLN from a wt vaccinated mouse could not be collected on day 38 due to technical problems. N=4-5, *: P<0.05, **: P<0.01 - Mann-Whitney U test.

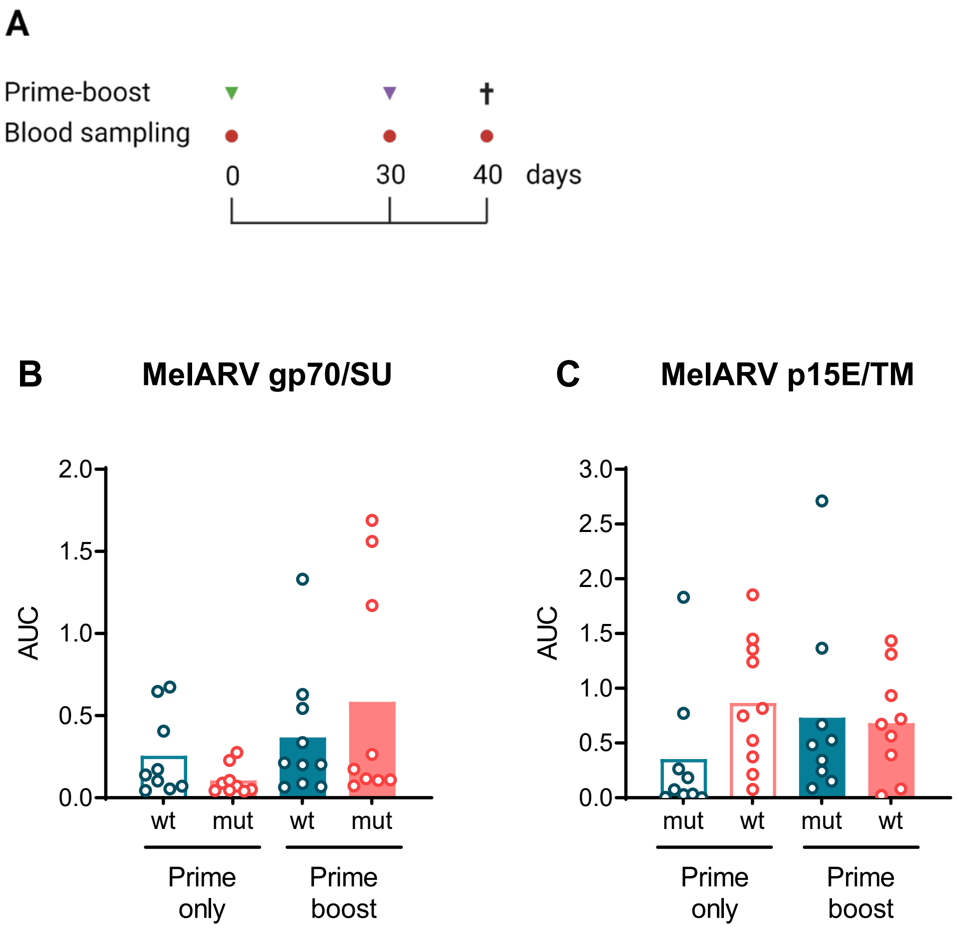

Figure S5. MelARV Env specific antibodies were detected in moderate levels in the serum of BALB/c vaccinated mice. Analysis of MelARV Env specific antibodies in mouse serum upon Ad19a/64-ERV ISDwt or ISDmut +/- Ad5-ERV ISDwt or ISDmut vaccination. (**A**) Schematic representation of the vaccination regimen and blood sampling. BALB/c mice were vaccinated (primed) s.c. (right paw) with either Ad19a/64-ERV ISDwt or Ad19a/64-ERV ISDmut. 30 days later, all mice were boosted s.c. (left paw) with either the Ad5-ERV ISDwt or with the Ad5-ERV ISDmut vaccine. Blood samples were taken from mice on days 0, 30 and 40. Generation of vaccine-induced MelARV-specific antibodies was assessed by measuring binding to MelARV Env proteins by ELISA. (**B**) Analysis of vaccine-induced antibody binding against MelARV Env gp70/SU subunit. (**C**) Analysis of vaccine-induced antibody binding against MelARV Env p15E/TM subunit. N=9-10. These data are a combination of two repeated independent experiments.

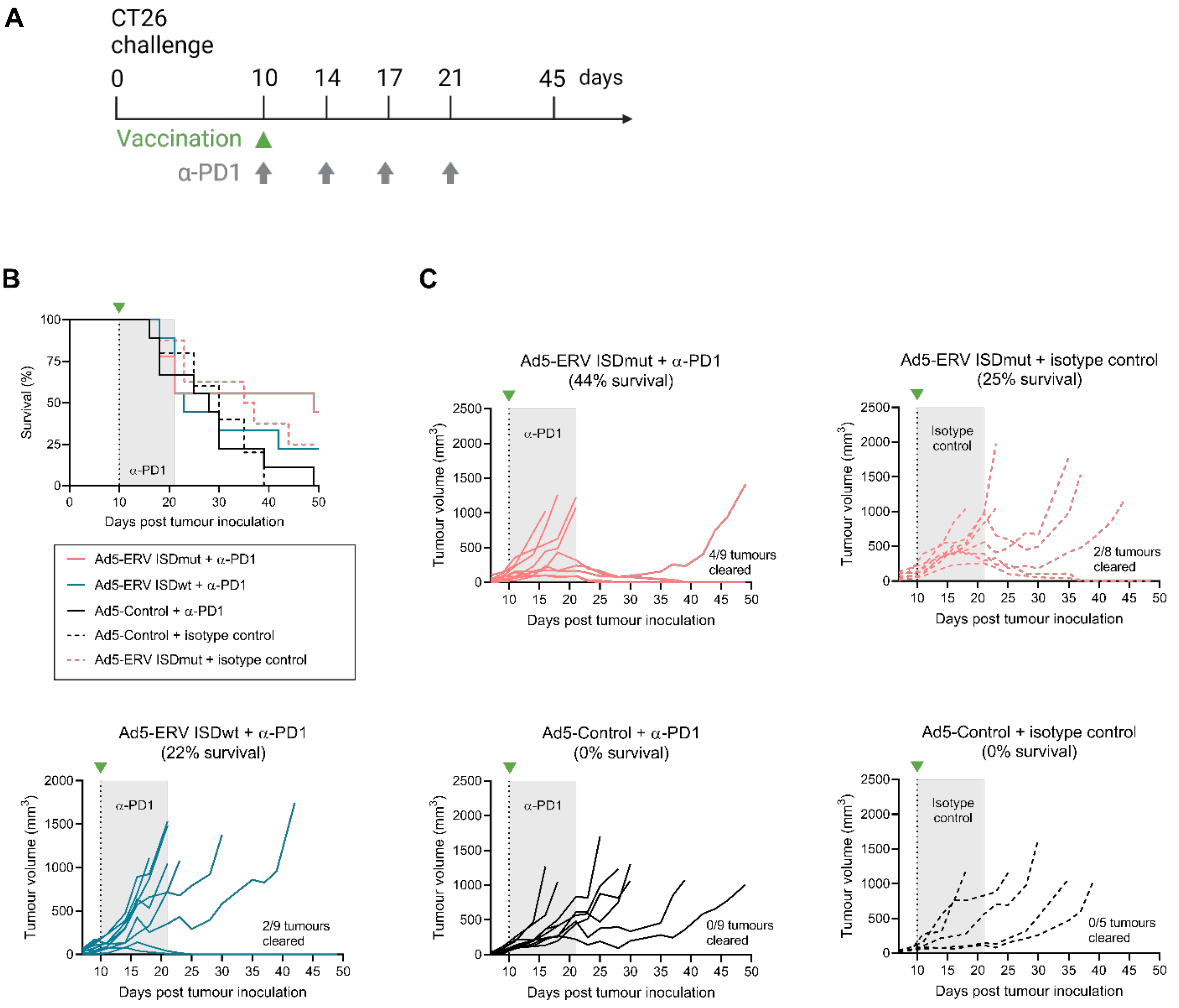

Figure S6. Ad5-ERV ISDmut synergises with α-PD1 checkpoint inhibitor and eradicates established colorectal tumours in mice. (**A**) Schematic representation of the tumour challenge and the therapeutic treatment. BALB/c mice were challenged s.c. (right flank) with 5×10^5 CT26 cells and tumours were measured every 2–3 days. Mice were distributed and vaccinated s.c. (left paw) on day 10 with either the Ad5-ERV ISDwt (blue), the Ad5-ERV ISDmut (coral) or the Ad5-Irrelevant (black) vaccine. α-PD1 or control antibodies (showed in dashed lines) were administered i.p. concomitant to the vaccination, and then three more times every 3-4 days. (**B**) Fraction of surviving mice post tumour challenge treated with Ad5 vaccines and α-PD1 antibodies. (**C**) Measurement of tumour volume (mm^3) over time after challenge and treatment. This experiment is a repeat of two independent experiments. N=5-9, *: $P<0.05$, **: $P<0.01$ - Log-rank (Mantel-Cox) test.
